# Continuous Measurement of Spatially Resolved Red Blood Cell Aggregation Using Multiple Side-Branch Channels

**DOI:** 10.3390/bios16090505

**Published:** 2026-09-08

**Authors:** Minjae Kim, Yang Jun Kang

**Affiliations:** Department of Mechanical Engineering, Chosun University, 10, Chosundae 1-gil, Dong-gu, Gwangju 61452, Republic of Korea; kmj129362@chosun.ac.kr

**Keywords:** spatiotemporal RBC aggregation map, multiple side-branch channels, continuous micro-blood flow, storage lesion

## Abstract

Red blood cell (RBC) aggregation is an important hemorheological property that influences blood viscosity, microcirculation, and stored-blood quality. However, conventional measurements commonly require repeated flow cessation or flow-rate modulation, limiting continuous monitoring and providing little information on spatial heterogeneity. This study presents a microfluidic platform for the spatiotemporal mapping of RBC aggregation during continuous blood flow. Multiple high-resistance side chambers connected to a main channel create low-shear-rate regions for aggregation while maintaining high shear in the main channel for RBC disaggregation. Flow rates and shear rates are evaluated using a hydraulic circuit model, numerical simulation, and micro-PIV measurements. An aggregation index (AI) map is introduced to quantify spatial and temporal changes in the side chambers. AI remains high and stable at flow rates of 0.5~1 mL/h. RBC aggregation increases significantly at concentrations of 15 mg/mL or with more dextran solution (*C_dex_*). At the selected flow rate of 1 mL/h and *C_dex_* = 40 mg/mL, the 95% confidence interval of AI is 0.477~0.600 for the proposed method, compared with 0.364~0.460 for the previous method. The proposed AI value is approximately 30% higher than that obtained using the previous method. Moreover, AI decreases progressively during four weeks of RBC storage. These findings demonstrate continuous and multiple-location detection of RBC aggregation and support the use of this platform for assessing hemorheological alterations and storage-induced RBC deterioration.

## 1. Introduction

Red blood cell (RBC) aggregation is a major determinant of blood viscosity under low-shear conditions, and pathologically enhanced aggregation can increase microvascular flow resistance and compromise tissue perfusion [1,2]. Under stasis or extremely low shear rates, RBCs reversibly form rouleaux and larger three-dimensional aggregates. RBC aggregation is governed by the combined effects of plasma macromolecules (fibrinogen, immunoglobulins), intrinsic cellular properties (surface charge, membrane–cytoskeletal organization, deformability, and morphology), and cellular age [1,3,4]. RBC aggregation measurements can be markedly affected by hematocrit (Hct), plasma viscosity, shear rate, temperature, and storage duration [5,6,7]. Excessive RBC aggregation increases blood viscosity under low-shear conditions and alters RBC partitioning at vascular bifurcations, potentially increasing flow resistance and causing heterogeneous microvascular perfusion and tissue oxygen delivery [1,8,9,10]. Abnormal RBC aggregation has been reported in diabetes [11], sepsis [12], obesity, cardiovascular disease [13,14], and sickle cell disease [15], highlighting its association with systemic inflammation and impaired microcirculation. Therefore, RBC aggregation measurement provides a potential biomarker for evaluating systemic inflammation, microcirculatory dysfunction, disease progression, and therapeutic effectiveness [1,16,17].

Conventional methods typically apply high shear to disperse existing RBC aggregates and then stop or slow the rotating system to allow for reaggregation. Aggregation is subsequently quantified from time-dependent changes in light transmission or backscatter [5,18]. A major limitation of conventional rotational aggregometers is their use of closed-chamber Couette flow, which does not reproduce the pressure-driven transport and spatially varying shear conditions of microvascular blood flow [19,20].

Microfluidic chips have emerged as useful platforms for RBC aggregation analysis because they require small sample volumes and permit precise control of microvascular-like, pressure-driven flow [21,22,23,24]. Some systems measured RBC aggregation using laser backscatter after vibration-induced dispersion, while others used pulsatile microchannel flow to determine the critical shear stress for aggregation [25]. Direct microscopic imaging and image-processing techniques have been used to quantify RBC aggregate size and spatial distribution under controlled microchannel flow conditions [9,26,27]. Furthermore, speckle-based analysis has sensitively detected increased RBC aggregation in diabetic blood [28], whereas bifurcating microchannels have characterized spatial differences in aggregate size, transport, and partitioning that conventional bulk aggregometers cannot resolve [9]. Microfluidic platforms have enabled the combined measurement of RBC aggregation with blood viscosity, pressure, or erythrocyte sedimentation rate. In parallel, low-cost miniaturized systems have demonstrated the potential for portable RBC aggregation testing [22,24,29,30]. However, many existing devices still apply high shear to break up RBC aggregates and then stop, slow, or periodically change the flow to allow them to form again. More recently, our group proposed a continuous-flow method for measuring RBC aggregation by allowing blood to flow from a high-shear main channel into high-resistance side-branch channels. The increased flow resistance reduces the flow rate and shear rate within these channels, allowing RBC aggregates to form without interrupting blood flow [31]. However, the previous method measured RBC aggregation only at a single location and could not capture its spatial and temporal heterogeneity. Therefore, simultaneous multi-site measurement of the spatiotemporal aggregation index is required.

In this study, to address the limitations of the previous method, the present method enables the simultaneous spatiotemporal mapping of non-uniform RBC aggregation without interrupting blood flow. The proposed microfluidic device consists of a single inlet, a single outlet, a main channel, and three side-branch channels, each equipped with an enlarged side chamber. The design concept relies on a strong contrast in hydraulic resistance between the main channel and the side-branch channels. The narrow passages connected to each side chamber impose high flow resistance, thereby diverting only a small fraction of blood into the branches and markedly reducing the local velocity and shear rate. Meanwhile, the main channel preserves a relatively high-shear environment that maintains RBC disaggregation. This shear-rate separation creates low-shear zones for RBC reaggregation within the side chambers while continuous flow is maintained throughout the system. Blood is continuously supplied at a constant flow rate using a syringe pump. The high shear rate in the main channel fully disaggregates RBCs, whereas the enlarged side chambers reduce the local shear rate to below 100 s^−1^, allowing for heterogeneous RBC aggregation to occur. Local aggregation indices are calculated by comparing the image intensity of each region in the side chambers with the mean intensity of the non-aggregated RBCs in the main channel.

Compared with conventional methods, the proposed method has several advantages. It measures RBC aggregation continuously without stopping the blood flow or changing the flow rate. It also shows how RBC aggregation differs across the side chambers and changes over time. RBC aggregation can be measured simultaneously in three side chambers, which improves the efficiency and reliability of measurements. In addition, the image intensity in the main channel, where RBCs are fully disaggregated, is used as a reference to measure local aggregation. The simple single-inlet and single-outlet design requires only a syringe pump operating at a constant flow rate.

## 2. Materials and Methods

### 2.1. Experimental Setup for Spatiotemporal Mapping of RBC Aggregation

**Figure 1:**
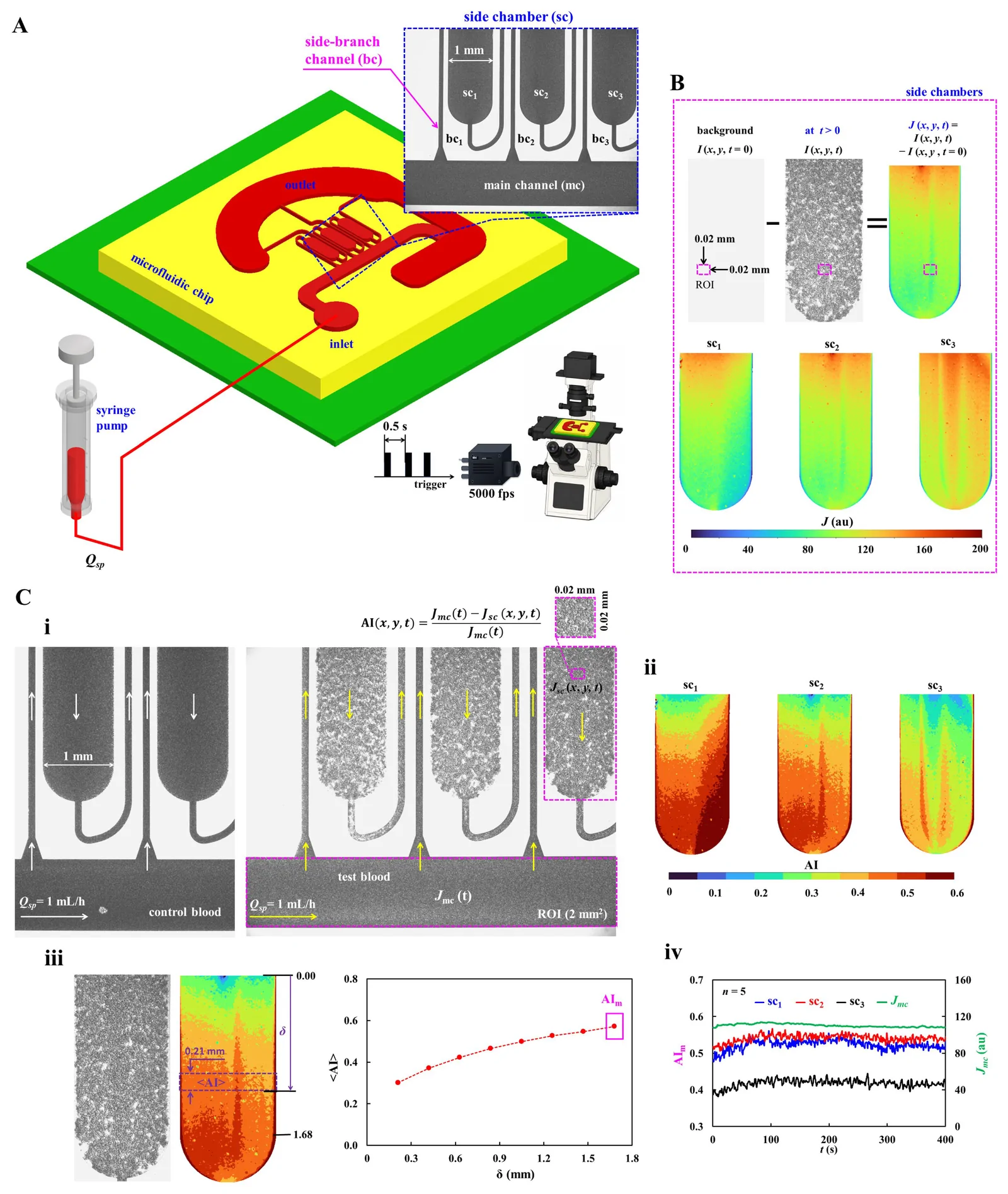
A proposed method for assessing time-lapse RBC aggregation index (AI) map in microfluidic channels. (**A**) Experimental setup, including a microfluidic chip, a syringe pump, and image acquisition system. The microfluidic channel was composed of a single inlet, a single outlet, a main channel (mc), and three side-branch channels, each with an enlarged side chamber (sc_1_, sc_2_, and sc_3_). The syringe pump was set to constant flow rate (*Q_sp_*). The image acquisition system was composed of an inverted microscope (4×, NA = 0.1), a high-speed camera (5000 fps), and an external trigger (pulse period: 0.5 s). (**B**) Quantification of blood imaging intensity inside each side chamber. A compensated image intensity (*J*) was calculated by subtracting a captured blood image (*I* [*x*, *y*, *t* > 0]) from background grayscale image (*I* [*x*, *y*, *t* = 0]). The lower panels exhibit image intensity distributions in three side chambers. (**C**) Calculation of AI and representative index (AI_m_). (**i**) Calculation of spatiotemporal AI map. The left panel shows a microscopic image of control blood. Arrow (‘→’) indicated the direction of blood flow. The right panel presents a microscopic image of test blood. (**ii**) The spatial distribution of AI in each side chamber. (**iii**) Determination of the representative value of spatially distributed AI. The left panel shows a bright-field image and its corresponding AI map, respectively. The maximum value of <AI> was then regarded as a representative value of AI (AI_m_). (**iv**) Time-lapse AI_m_ with respect to each side chamber (sc_1_, sc_2_, and sc_3_) and *J_mc_*.

As shown in Figure 1A, the experimental setup consisted of a microfluidic chip, a syringe pump (neMESYS, CETONI GmbH, Korbussen, Germany), and an image acquisition system. The microfluidic channel was composed of a single inlet, a single outlet, a main channel (MC: width = 1 mm; length = 14.48 mm), and three side-branch channels (BCs), each containing an enlarged side chamber (sc_1_, sc_2_, and sc_3_). Each side-branch channel consisted of three segments connected in series: a front narrow channel (width = 0.1 mm’ length = 5.23 mm), a side chamber (width = 1 mm; length = 3 mm), and a rear narrow channel (width = 0.1 mm). The rear-channel lengths were 8.915, 8.865, and 7.61 mm for BC_1_, BC_2_, and BC_3_, respectively. The spacing between adjacent side-branch channels was fixed at 1.6 mm. All channels had a uniform depth of 0.05 mm.

A silicon master mold was fabricated from a four-inch silicon wafer (MTI Korea, Seoul, South Korea). The microchannel pattern was defined using conventional photolithography. The patterned wafer was then etched by deep reactive ion etching (DRIE) to form the mold features [32]. For PDMS replica molding, the Sylgard 184 base and curing agent (Dow Corning, Midland, MI, USA) were mixed at a mass ratio of 10:1. The uncured mixture was degassed under vacuum for 1 h and then cured at 65 °C for 2 h. After curing, the PDMS replica was carefully removed from the silicon master and cut to the desired dimensions. An inlet port was formed with a punch with an outer diameter of 2 mm, while a larger-sized outlet port was punched to encompass the downstream ends of the main and side-branch channels. The PDMS layer and glass substrate were activated by oxygen plasma using a plasma treatment system (CUTE-MPR, Femto Science Co., Ltd., Hwaseong, Republic of Korea) and then brought into contact to form an irreversible bond. To strengthen the PDMS–glass bond, the assembled microfluidic chip was heated at 120 °C for 10 min.

One end of a polyethylene tube (inner diameter = 0.25 mm; length = 300 mm) was connected to a syringe needle attached to a 1 mL disposable syringe, while the other end of the tubing was inserted into the inlet port of the microfluidic device.

To reduce nonspecific protein adsorption, the microchannels were treated with a 0.2% bovine serum albumin (BSA) solution for 10 min. Before introducing the blood sample, the channels were rinsed with 1× phosphate-buffered saline (PBS) to remove the remaining BSA solution. Test blood (0.2~0.3 mL) was loaded into a disposable syringe, which was mounted on a syringe pump. The test blood was infused at a constant flow rate of *Q_sp_*.

The microfluidic chip was placed on an inverted microscope (IX81, Olympus, Tokyo, Japan) fitted with a 4× objective lens (NA = 0.1). Blood-flow images were recorded at 5000 frames per second (fps) using a high-speed camera (FASTCAM MINI, Photron, Tokyo, Japan). Image recording was initiated at 0.5 s intervals by a function generator. All experiments were conducted at room temperature (25 °C).

### 2.2. Quantification of Blood Imaging Intensity in the Side Chambers

As shown in Figure 1B, the initial image of each side chamber, *I* (*x*, *y*, *t* = 0), was used as the background reference. Each subsequent image, *I* (*x*, *y*, *t*) for *t* > 0, was subtracted from the corresponding reference image to obtain a difference image: *J* (*x*, *y*, *t*) = *I* (*x*, *y*, *t* = 0) − *I* (*x*, *y*, *t* > 0). The difference images were analyzed using MATLAB R2025b (MathWorks, Natick, MA, USA). A fixed local region of interest (ROI, 0.02 × 0.02 mm^2^) within each side chamber was defined to calculate the mean image intensity. The three lower panels show the image intensity distributions measured in the three side chambers (sc_1_, sc_2_, and sc_3_). The test blood was prepared by suspending normal RBCs into a dextran solution (30 mg/mL).

### 2.3. Spatiotemporal Mapping of RBC Aggregation Index

In this study, spatiotemporal maps of the RBC aggregation index were generated by comparing the image intensity of fully disaggregated RBCs in the main channel with that of aggregated RBCs in each side chamber. Herein, to demonstrate the operating principle of the proposed method, control and test blood samples (Hct = 50%) were prepared by suspending normal RBCs into 1× PBS and dextran solution (40 mg/mL), respectively. Each blood was infused into a microfluidic channel at a constant flow rate of *Q_sp_* = 1 mL/h.

In Figure 1C(i), blood flow images illustrate the differences in RBC aggregation between control blood and test blood. For the control blood (left-side panel), the image intensity remained relatively uniform throughout all microfluidic channels, indicating negligible RBC aggregation. Several arrows (‘→’) indicate the direction of blood flow. In contrast, for the test blood (right-side panel), RBC aggregation occurred in each side chamber, resulting in a substantial decrease in image intensity. However, no noticeable RBC aggregation was observed in the main channel.

To construct a spatiotemporal RBC aggregation map, AI = AI (*x*, *y*, *t*), a region of interest (ROI, 2 mm^2^) was selected in the main channel as the reference region. The mean image intensity within this ROI was calculated as *J_mc_*(*t*) and used as the reference intensity, representing RBCs under high-shear, disaggregated conditions. The local image intensity at each position in the side chambers was denoted by *J*_sc_ (*x*, *y*, *t*). Because RBC aggregation in the side chambers reduced the local image intensity relative to the main-channel reference, the normalized intensity difference was used to quantify RBC aggregation. Accordingly, the spatiotemporal aggregation index was defined as

(1) $$AI\left(x,y,t\right)=\frac{J_{mc}\left(t\right)-J_{sc}\left(x,\;\;y,\;t\right)}{J_{mc}\left(t\right)}$$

A larger AI (*x*, *y*, *t*) value therefore indicates a greater degree of local RBC aggregation. As shown in Figure 1C(ii), the AI exhibited a nonuniform spatial distribution within each side chamber at a given time and gradually increased along the direction of blood flow.

Next, a representative value was defined to characterize the spatially distributed AI within each side chamber. In Figure 1C(iii), the left panel presents the bright-field image and its corresponding AI map. The spatially averaged AI, <AI>, was calculated within a specific ROI (0.21 mm^2^) positioned along the flow direction. The right panel showed <AI> as a function of downstream distance (*δ*). The *δ* was defined using the upstream edge of the acquired microscopic image as the reference position and measured along the direction of blood-flow. The <AI> value gradually increased with respect to *δ* and reached its maximum at *δ* = 1.68 mm. This maximum value was therefore selected as the representative aggregation index for each side chamber and denoted as AI_m_. As shown in Figure 1C(iv), the time-dependent AI_m_ was determined for each side chamber (sc_1_, sc_2_, and sc_3_) and *J_mc_*. Measurements were repeated five times using the test blood (*n* = 5), and each data point represents the mean of the five independent experiments. Within the 95% confidence interval, the AI_m_ ranges were 0.520~0.584 for sc_1_, 0.541~0.605 for sc_2_, and 0.417~0.481 for sc_3_, respectively. Furthermore, *J_mc_* remained nearly constant throughout the measurement period, ranging from 109.7 to 109.9 a.u.

Because the proposed method enables RBC aggregation to be measured without interrupting blood flow, AI_m_ could be continuously monitored as a function of time.

### 2.4. Blood Sample Preparation

Concentrated RBCs were purchased from the Gwangju–Chonnam Blood Bank (Gwangju, Republic of Korea) and stored under refrigerated conditions until use. Following a previously established washing protocol [33], normal RBCs were isolated through repeated removal of the supernatant and buffy coat. Test blood was then prepared by suspending normal RBCs into a specific medium (1× PBS, dextran solution).

First, test blood with enhanced RBC aggregation was prepared by suspending normal RBCs into dextran solutions (*C_dex_* = 5~40 mg/mL). The dextran solutions were prepared by dissolving dextran powder derived from *Leuconostoc* spp. (molecular weight: 450–650 kDa, Sigma-Aldrich, St. Louis, MO, USA) into 1× PBS. The hematocrit of each test blood was adjusted to 50%.

Second, to investigate the effect of storage duration on RBC aggregation, concentrated RBCs obtained from the blood bank were stored under refrigerated conditions for 0, 1, 2, 3, and 4 weeks. At each storage interval, test blood was prepared by resuspending the stored RBCs into dextran solutions with *C_dex_* ranging from 15 to 40 mg/mL. The hematocrit of all samples was adjusted to 50%.

### 2.5. Statistical Analysis

Statistical analyses were performed using Minitab 22.4 (Minitab LLC, State College, PA, USA) and Microsoft Excel 365 (Microsoft Corporation, Redmond, WA, USA). Assuming normally distributed data, the results are reported as the mean ($\bar{x}$) ± standard deviation (σ), with *n* indicating the number of data points. Unless otherwise specified, all error bars represent the standard deviation. Two-sided 95% confidence intervals were estimated as $\bar{x}\;\pm \;1.96\frac{\;\sigma }{\sqrt{n}}$. Differences among multiple groups were evaluated by one-way ANOVA. Statistical significance was accepted at *p* < 0.05. Linear regression was performed in Microsoft Excel, and the coefficient of determination (R^2^) and *p* were used to evaluate the goodness of fit and statistical significance, respectively.

## 3. Results and Discussion

### 3.1. Estimation of Flow Rates and Shear Rates in the Main Channel and Side Chambers

Previous studies have demonstrated that RBC aggregation is promoted under low-shear conditions and progressively decreases as the shear rate increases [1,34,35]. Accordingly, shear rates below approximately 100 s^−1^ were considered suitable for inducing RBC aggregation in the present microfluidic device [20,31,36,37]. Because the test blood was supplied at a constant flow rate, *Q_sp_*, the supplied flow rate strongly affected the shear-rate distributions throughout the microfluidic channels. Therefore, the shear-rate distributions were quantitatively evaluated using three complementary approaches: a discrete fluidic-circuit model, a full-scale finite-element simulation (i.e., computational fluid dynamics [CFD]), and experimental micro-particle image velocimetry (micro-PIV). For convenience, a 30% glycerin solution as Newtonian fluid was selected as the test fluid. Based on values reported in a previous study [38], its dynamic viscosity and density were assumed to be *μ* = 2.5 cP and *ρ* = 1080 kg/m^3^, respectively.

**Figure 2:**
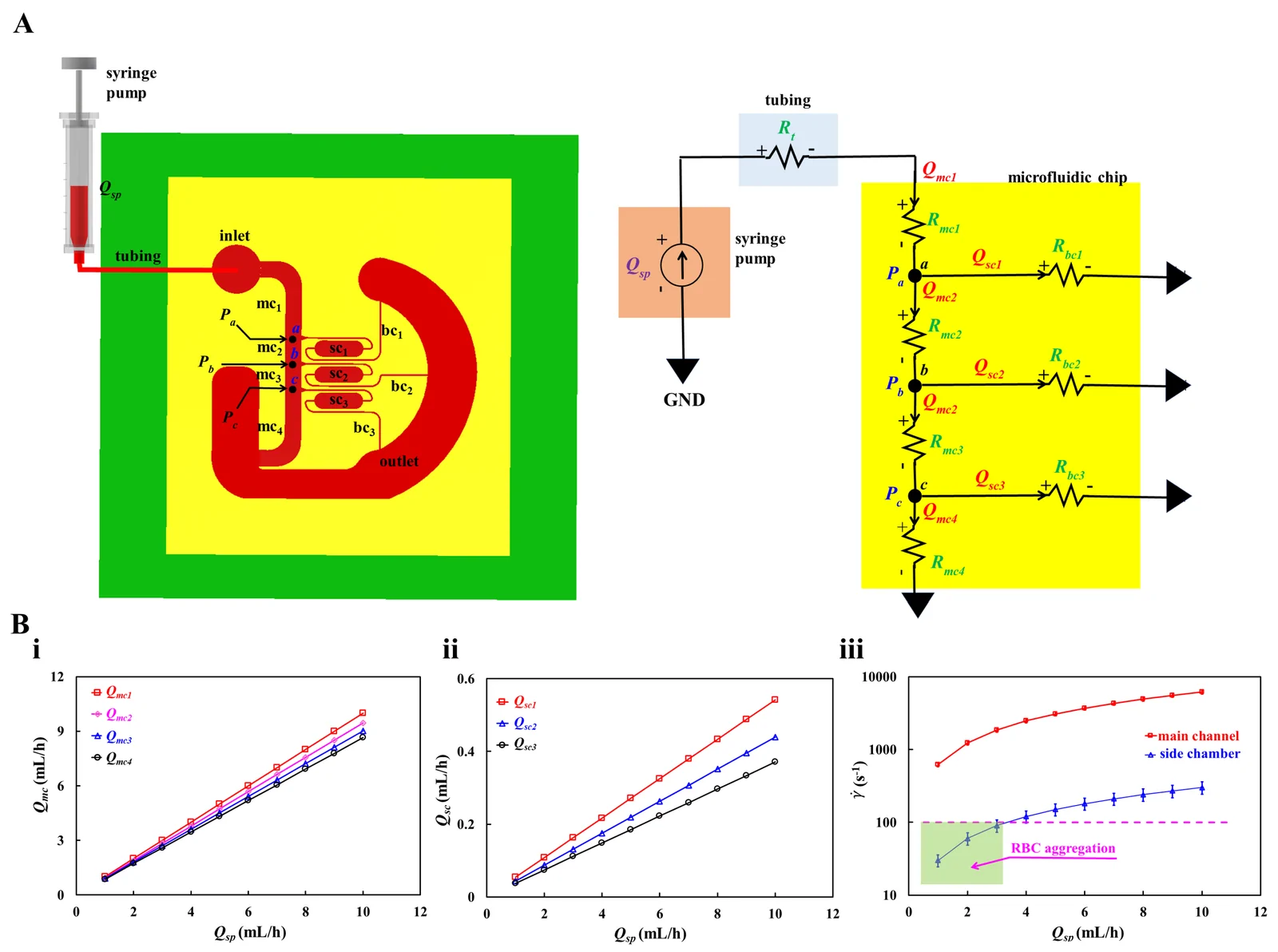
Determination of shear rate ($\dot{\gamma }$) using a discrete circuit model. (**A**) A fluidic circuit model for determining flow rate and shear rate in the microfluidic channels. The left-side panel shows a schematic diagram of the microfluidic system, such as a syringe pump, tubing, and a microfluidic chip. The main channel is divided into four segments (mc_1_, mc_2_, mc_3_, and mc_4_). Three bifurcations are denoted as bc_1_, bc_2_, and bc_3_. The side chambers are denoted as sc_1_, sc_2_, and sc_3_. The pressures at each junction (*a*, *b*, *c*) are denoted as *P_a_*, *P_b_*, and *P_c_*. The right panel presents a fluidic-circuit model of the proposed microfluidic system. The fluidic-circuit model consists of the flow-rate source (*Q_sp_*), and several hydraulic resistances. A symbol, ▼, indicates zero value of gauge pressure (i.e., GND). Flow rate of each channel is denoted as *Q_mc_*_1_~*Q_mc_*_4_ (main channel) and *Q_sc_*_1_~*Q_sc_*_3_ (side chamber). (**B**) Estimation of flow rates and shear rates as a function of *Q_sp_*. (**i**) Variations in *Q_mc_*_1_, *Q_mc_*_2_, *Q_mc_*_3_, and *Q_mc_*_4_ as a function of *Q_sp_*. (**ii**) Variations in *Q_sc_*_1_, *Q_sc_*_2_, and *Q_sc_*_3_ as a function of *Q_sp_*. (**iii**) Variations in $\dot{\gamma }$ in the main channel and side chamber with respect to *Q_sp_*. The light-green box indicates the allowable range of infused flow rate for inducing RBC aggregation in side chambers.

First, as shown in Figure 2A, a discrete fluidic-circuit model was constructed to estimate the flow rates and shear rates throughout the proposed microfluidic system at a prescribed syringe-pump flow rate, *Q_sp_*. The left-side panel illustrates the experimental setup, consisting of a syringe pump, a connecting tubing, and a microfluidic chip. The main channel was divided by three bifurcation points into four segments, denoted as mc_1_, mc_2_, mc_3_, and mc_4_. The three side-branch channels and corresponding side chambers were designated as bc_1_~bc_3_ and sc_1_~sc_3_, respectively. The pressures at junctions *a*, *b*, and *c* were denoted as *P_a_*, *P_b_*, and *P_c_*, respectively. The syringe pump delivered the test blood at a constant flow rate, *Q_sp_*, through tubing connecting the syringe needle to the inlet of the microfluidic chip. The right panel illustrates the equivalent fluidic-circuit model of the proposed microfluidic system. The model comprised a constant flow-rate source, *Q_sp_*, and the hydraulic resistances of the tubing (*R_t_*), main-channel segments (*R_mc_*_1_*~R_mc_*_4_), and side-branch channels (*R_bc_*_1_~*R_bc_*_3_). The ground symbol (▼) represents the reference pressure, corresponding to a zero value of gauge pressure. The flow rates through the main-channel segments and side chambers are denoted by *Q_mc_*_1_~*Q_mc_*_4_ and *Q_sc_*_1_~*Q_sc_*_3_, respectively. By applying the conservation of mass to the steady flow at junctions (*a*, *b*, and *c*), where the total inflow equals the total outflow at each junction, the following three equations were obtained:

(2) $$Q_{sp}\;=\;\frac{P_{a}}{R_{bc1}}\;+\;\frac{P_{a}\;-\;P_{b}}{R_{mc2}}$$

(3) $$\frac{P_{a}-P_{b}}{R_{mc2}}=\frac{P_{b}}{R_{bc2}}+\frac{P_{b}-{\;P}_{c}}{R_{mc3}}$$

(4) $$\frac{P_{b}-P_{c}}{R_{mc3}}=\frac{P_{c}}{R_{bc3}}+\frac{P_{c}}{R_{mc4}}$$

Simultaneous solution of the three governing equations yielded the junction pressures (*P_a_*, *P_b_*, and *P_c_*). The resulting analytical expressions were given by

(5) $$P_{a}=\frac{\alpha }{\frac{\alpha }{R_{bc1}}+\frac{\alpha -\beta }{R_{mc2}}}Q_{sp}$$

(6) $$P_{b}=\frac{\beta }{\frac{\alpha }{R_{bc1}}+\frac{\alpha -\beta }{R_{mc2}}}Q_{sp}$$

(7) $$P_{c}=\frac{1}{\frac{\alpha }{R_{bc1}}+\frac{\alpha -\beta }{R_{mc2}}}Q_{sp}$$

The dimensionless coefficients α and *β* appearing in Equations (5)–(7) were defined as

(8) $$\alpha =\left(1+\frac{R_{mc2}}{R_{bc2}}\right)\left(1+\frac{R_{mc3}}{R_{bc3}}+\frac{R_{mc3}}{R_{mc4}}\right)+(\frac{R_{mc2}}{R_{bc3}}+\frac{R_{mc2}}{R_{mc4}})$$

(9) $$\beta =(1+\frac{R_{mc3}}{R_{bc3}}+\frac{R_{mc3}}{R_{mc4}})$$

Using the analytically determined junction pressures, the flow rates through the main-channel segments (*Q_mc_*_1_~*Q_mc_*_4_) and side chambers (*Q_sc_*_1_~*Q_sc_*_3_) were calculated using the Hagen–Poiseuille equation (i.e., pressure drop = fluidic resistance × flow rate) [39]. As shown in Figure 2B, flow rates and shear rates in the microfluidic channels were calculated as a function of infused flow rate (*Q_sp_*). For convenience, using the fluid property of 1× PBS and channel dimensions, the hydraulic resistances of the four main channel segments were calculated as *R_mc_*_1_ = 0.54, *R_mc_*_2_ = 0.15, *R_mc_*_3_ = 0.15, and *R_mc_*_4_ = 0.54 TPa×s/m^3^. The corresponding hydraulic resistances of the three side-branch channels were *R_bc_*_1_ = 13.87, *R_bc_*_2_ = 13.82, and *R_bc_*_3_ = 12.61 TPa×s/m^3^. Based on these resistance values, coefficients *α* and *β* were calculated to be 1.606 and 1.296, respectively. The substantially higher resistance of the side-branch channels limited the branch flow and produced a low-shear environment within the enlarged side chambers. As shown in Figure 2B(i), the flow rates through the four main channel segments (*Q_mc_*_1_~*Q_mc_*_4_) were determined as functions of the imposed flow rate, *Q_sp_*, which ranged from 1 to 10 mL/h. Similarly, Figure 2B(ii) presents the flow rates through the three side chambers (*Q_sc_*_1_~*Q_sc_*_3_) as functions of *Q_sp_*. As expected, the flow rate in each upstream channel was higher than that in the corresponding downstream channel. Furthermore, the flow rate in the main channel was approximately 20 times higher than that in each side chamber.

Figure 2B(iii) shows the shear rates, $\dot{\gamma }$, in the main channel and side chambers as functions of *Q_sp_*. At each *Q_sp_*, the mean shear rate in the main channel was calculated from the values obtained for its four segments (mc_1_~mc_4_), whereas that in the side chambers was calculated from the values for sc_1_~sc_3_. Accordingly, each data point is presented as the mean ± standard deviation, where the standard deviation reflected the variations among the respective channel sections. The results confirmed that RBC aggregation was effectively suppressed in the main channel at *Q_sp_* > 1 mL/h, where the shear rate remained above 100 s^−1^. However, the shear rate in the side chambers remained below 100 s^−1^ when *Q_sp_* < 3 mL/h. The light-green shaded region indicates the flow-rate range considered suitable for inducing RBC aggregation in the side chambers.

**Figure 3:**
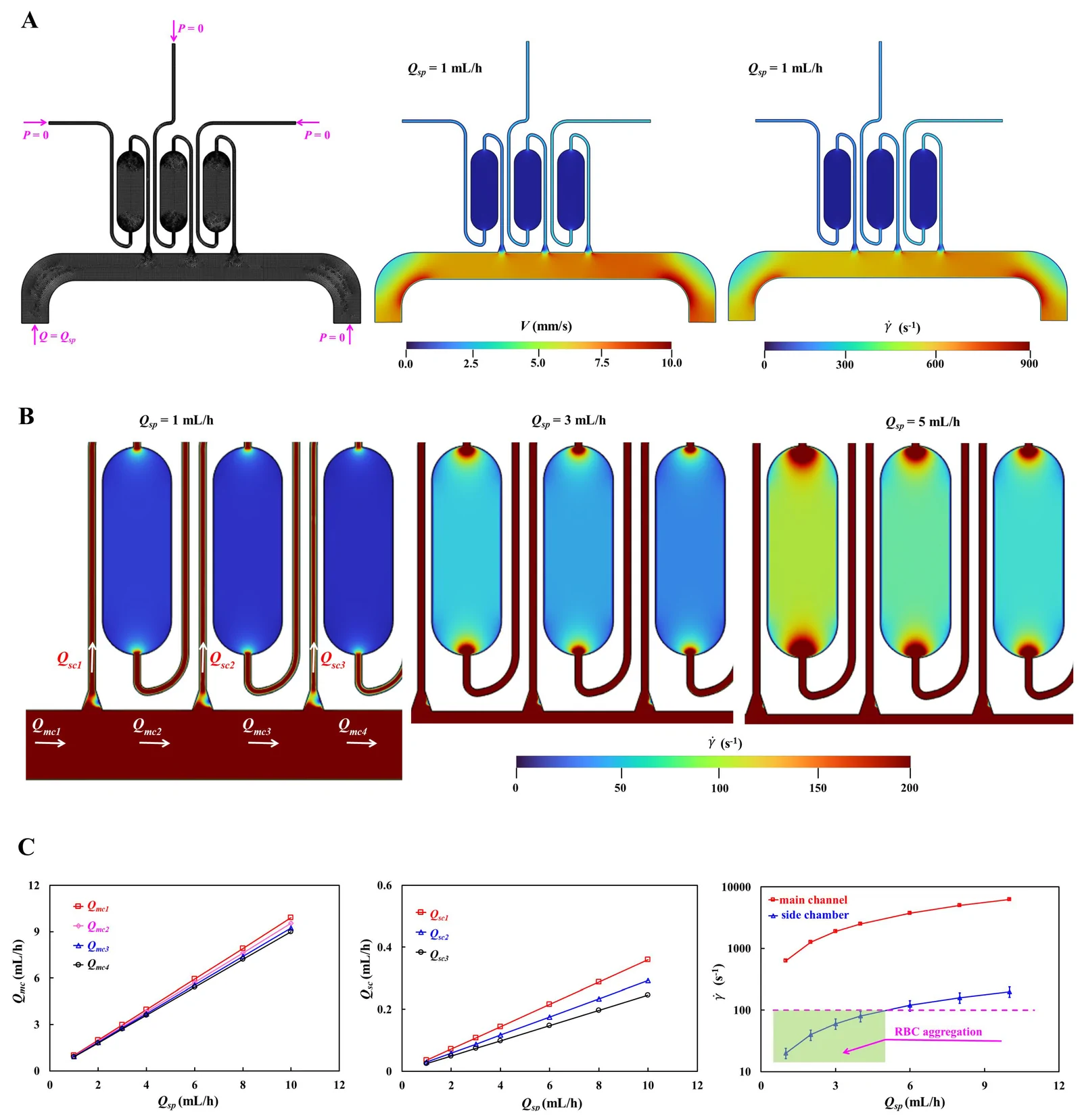
Estimation of shear rate ($\dot{\gamma }$) using a full-scale computational fluid dynamics simulation of the proposed microfluidic chip. (**A**) Distributions of velocity and shear rate obtained using commercial software (COMSOL Multiphysics). The left panel shows the meshes generated for the microfluidic channels. The middle panel shows velocity distributions at the channel midplane, where *Q_sp_* = 1 mL/h. The right panel represents shear rate distributions at the bottom wall at *Q_sp_* = 1 mL/h. (**B**) Distributions of shear rate as a function of *Q_sp_* = 1, 3, and 5 mL/h. (**C**) Estimation of flow rate and shear rate as a function of *Q_sp_*. The left-side panel shows variations in flow rate in the main channel (*Q_mc_*_1_~*Q_mc_*_4_) as a function of *Q_sp_*. The middle panel shows variations in flow rates in side chambers (*Q_sc_*_1_~*Q_sc_*_3_) as a function of *Q_sp_*. The right-side panel exhibits variations in shear rates in the main channel and side chambers as a function of infused flow rate. The light-green box indicates the allowable range of infused flow rate for inducing RBC aggregation in side chambers.

Second, as shown in Figure 3A, a commercial computational fluid dynamics software (COMSOL Multiphysics, version 6.2, Burlington, MA, USA) was used to estimate the flow rates and shear rates in the microfluidic channels. The left panel shows the meshes generated for the microfluidic channels. A full-scale three-dimensional model of the channels was constructed using the three-dimensional laminar flow interface and discretized into 256,580 hexahedral elements. A constant flow rate was prescribed at the inlet, while zero-pressure conditions (*p* = 0) were imposed at the four outlets. The steady-state flow was solved using a fully coupled nonlinear solver. At each nonlinear step, the resulting linear system was solved with GMRES and a smoothed-aggregation algebraic multigrid preconditioner. PARDISO was used at the coarsest level. A nonlinear tolerance of 10^−3^ and automatic damping were used to improve convergence. The model had approximately 1.2 × 10^6^ degrees of freedom, converged in four nonlinear iterations, and required about 131 s of computation time. As shown in the middle panel, at *Q_sp_* = 1 mL/h, the flow velocity at the channel midplane was substantially lower in the side chambers than in the main channel. The flow rate in each channel was estimated by multiplying its mean velocity by its cross-sectional area (*Q* = <*V*> × *A*, <*V*>: average velocity, and *A* = 0.05 mm^2^). The right panel shows the wall shear-rate distributions at the bottom surface of the microfluidic channels at *Q_sp_* = 1 mL/h. The shear rate was substantially lower in the side chambers than in the main channel. As shown in Figure 3B, the shear-rate distribution in the side chambers was examined over *Q_sp_* = 1~3 mL/h. Herein, the flow rates through the four main channel segments and three side chambers are denoted by *Q_mc_*_1_~*Q_mc_*_4_ and *Q_sc_*_1_~*Q_sc_*_3_, respectively. The CFD results showed a substantial reduction in flow velocity and shear rate within the enlarged side chamber, with no significant flow recirculation observed. Throughout this flow rate range, the shear rate in the wide region of each side chamber remained below 100 s^−1^, whereas it exceeded 100 s^−1^ in the narrow channels located before and after the chamber. These results indicated that RBC aggregation could be expected to occur primarily in the wide regions of the side chambers when *Q_sp_* ≤ 3 mL/h.

As shown in Figure 3C, the flow and shear rates in the main channel and side chambers were evaluated as functions of the supplied flow rate (*Q_sp_*). The left and middle panels show the flow rates in the main-channel segments (*Q_mc_*_1_~*Q_mc_*_4_) and side chambers (*Q_sc_*_1_~*Q_sc_*_3_), respectively. The flow rate was higher in the upstream main channel segments than in the downstream segments. Overall, the flow rates in the main channel were approximately 31.5 times those in the side chambers. The right panel shows the shear rates in the main channel and side chambers as functions of the supplied flow rate (*Q_sp_*). At *Q_sp_* = 1 mL/h, the shear rate in the main channel exceeded 100 s^−1^, which was sufficient to fully disaggregate the RBCs. In contrast, the shear rate in the side chambers remained below 100 s^−1^ when *Q_sp_* < 4 mL/h, allowing for RBC aggregation to occur. The light-green region indicates the suitable range of *Q_sp_* for inducing RBC aggregation in the side chambers.

**Figure 4:**
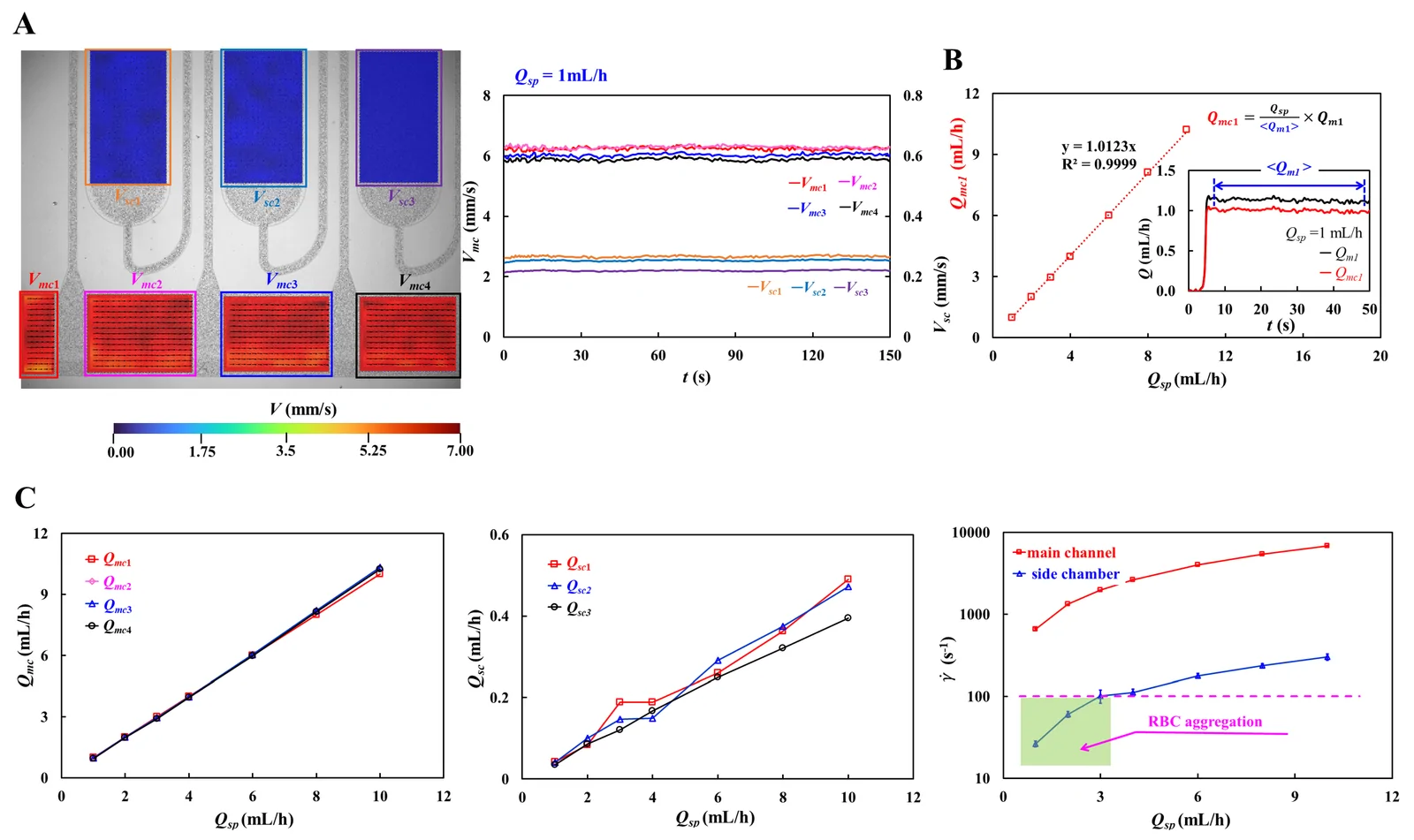
Experimental quantification of flow rate and shear rate in microfluidic channels using micro-PIV technique. Herein, using a syringe pump, a 30% glycerin solution containing 3% RBCs (i.e., fluid tracers) was supplied into a microfluidic chip at a constant flow rate of *Q_sp_*. (**A**) Determination of flow rate in main channel and side chamber as a function of *Q_sp_*. The left panel shows the velocity fields in the main channel (mc_1_~mc_4_) and side chamber (sc_1_~sc_3_) at *Q_sp_* = 1 mL/h. The middle panel presents the time-lapse flow rates in the main channel (i.e., mc_1_~mc_4_) and side chamber (sc_1_~sc_3_) at *Q_sp_* = 1 mL/h. Right panel shows variation in *Q_mc_*_1_ (the first segment in the main channel) as a function of *Q_sp_*. Inset showed time-lapse *Q_mc_*_1_ (corrected flow rate) and *Q_m_*_1_ (uncorrected flow rate). The corrected flow rate (*Q_mc_*_1_) was obtained as *Q_mc_*_1_ = *Q_sp_*/<*Q_m_*_1_> × *Q_m_*_1_. Herein, the <*Q_m_*_1_> denotes the mean of the uncorrected flow rate. (**B**) Variations in *Q_mc_*_1_ as a function of *Q_sp_*. Herein, the *Q_mc_*_1_ was corrected as *Q_mc_*_1_ = *Q_sp_*/<*Q_m_*_1_> × *Q_m_*_1_, where <*Q_m_*_1_> denotes the average flow rate of *Q_m_*_1_ over plateau intervals. (**C**) Variations in shear rate ($\dot{\gamma }$) in main channel and side chamber as a function of *Q_sp_*. The left-side panel shows variations in flow rate in the main channel (*Q_mc_*_1_, *Q_mc_*_2_, *Q_mc_*_3_, and *Q_mc_*_4_) with respect to *Q_sp_*. The middle panel shows variations in flow rate in the side chamber (*Q_sc_*_1_, *Q_sc_*_2_, and *Q_sc_*_3_) with respect to *Q_sp_*. The right-side panel presents variations in shear rate in the main channel and side chamber as a function of *Q_sp_*. The purple dashed line denotes the upper threshold of shear rate for inducing RBC aggregation.

Third, the predictions from the discrete circuit model and CFD simulation were experimentally validated using the micro-PIV technique [40,41,42,43]. Herein, a 30% glycerol solution containing 3% RBCs as flow tracers was infused at a constant flow rate, *Q_sp_*. As shown in Figure 4A, the micro-PIV technique was used to determine the flow rates in the main channel and side chambers as functions of *Q_sp_*. Specific ROIs were selected in four main channel segments (*mc*_1_~*mc*_4_) and three side chambers (sc_1_~sc_3_). Time-resolved micro-PIV analysis [43] was then performed to obtain the velocity fields within each ROI using 77 × 77 μm^2^ interrogation windows with 50% overlap. The mean velocity measured within each ROI was denoted as *V_mc_*_1_*~V_mc_*_4_ for the main-channel segments and *V_sc_*_1_~*V_sc_*_3_ for the side chambers. The right panel showed the time-dependent velocities in the main channel segments (*V_mc_*_1_~*V_mc_*_4_) and side chambers (*V_sc_*_1_~*V_sc_*_3_) at *Q_sp_* = 1 mL/h. The upstream segments exhibited higher velocities than the downstream segments.

Figure 4B shows the flow rate through the first main channel segment (*Q_mc_*_1_) as a function of the imposed flow rate (*Q_sp_*). The uncorrected flow rate was calculated from the mean velocity as *Q_m_*_1_ = *V_mc_*_1_ × *A*, where *A* is the cross-sectional area of the main channel. As shown in the inset, *Q_m_*_1_ was approximately 10% higher than the imposed flow rate at *Q_sp_* = 1 mL/h. Because *Q_sp_* was prescribed, the measured flow rate was corrected using *Q_mc_*_1_ = *Q_sp_*/<*Q_m_*_1_> × *Q_m_*_1_, where <*Q_m_*_1_> denotes the time-averaged uncorrected flow rate. After correction, the steady-state value of *Q_mc_*_1_ agreed with *Q_sp_*. The same correction factor (*Q_sp_*/<*Q_m_*_1_>) was applied to the flow rates measured in the remaining main channel segments and side chambers: *Q_mci_* = *Q_sp_*/<*Q_m_*_1_> × *Q_mi_* and *Q_scj_* = *Q_sp_*/<*Q_m_*_1_> × *Q_sj_*, where *i* = 1~4 and *j* = 1~3. *Q_sj_* denotes the uncorrected flow rate in side chamber. From the results, the corrected *Q_mc_*_1_ increased linearly with respect to *Q_sp_*, following *Q_mc_*_1_ = 1.0123 *Q_sp_* (R^2^ = 0.9999). As shown in Figure 4C, the flow rates in the main-channel segments and side chambers were evaluated as functions of the imposed flow rate, *Q_sp_*. The left panel presents *Q_mc_*_1_, *Q_mc_*_2_, *Q_mc_*_3_, and *Q_mc_*_4_, whereas the middle panel shows *Q_sc_*_1_, *Q_sc_*_2_, and *Q_sc_*_3_. The flow rate in every channel segment increased linearly with *Q_sp_*. Overall, the flow rates in the main channel segments were approximately 27-fold higher than those in the side chambers. The right panel shows the shear rates in the main-channel segments and side chambers as functions of the imposed flow rate (*Q_sp_*). At *Q_sp_* = 1 mL/h, the shear rates in the main channel exceeded 100 s^−1^. In contrast, the shear rates in the side chambers remained below 100 s^−1^ for *Q_sp_* < 3 mL/h. The purple dashed line indicates the upper shear-rate threshold for RBC aggregation.

**Figure 5:**
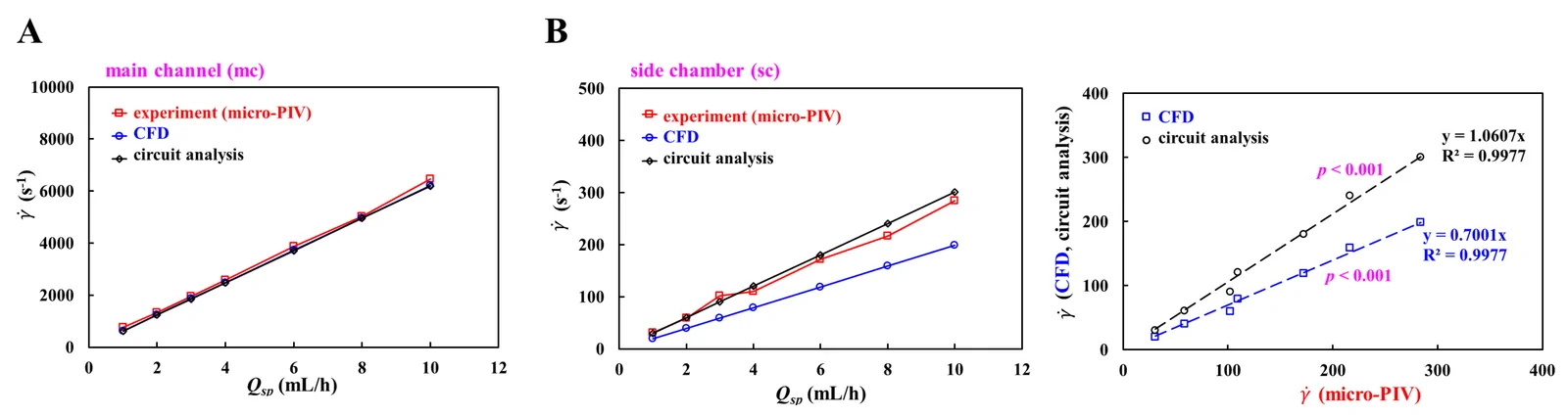
Quantitative comparison of shear rates obtained using three methods (i.e., circuit analysis, CFD, and experiment). (**A**) Variations in shear rate in the main channel obtained by three methods as a function of *Q_sp_*. (**B**) Variations in shear rate in the side chamber obtained by three methods as a function of *Q_sp_*. According to linear analysis, shear rates estimated by circuit analysis and CFD were linearly proportional to the experimental values.

Finally, the shear rates estimated using three independent methods, including fluidic-circuit analysis, computational fluid dynamics (CFD), and micro-PIV measurements, were compared over the investigated range of imposed flow rates (*Q_sp_*). As shown in Figure 5A, the shear rates in the main channel obtained using the three methods closely agreed and exhibited nearly identical linear increases with respect to *Q_sp_*.

The left panel of Figure 5B compares the shear rates in the side chambers. Although all three methods showed similar flow-rate dependent trends, the CFD predictions were consistently lower than the values obtained from the fluidic circuit analysis and micro-PIV measurements. The correlations between the methods are presented in the right panel of Figure 5B. Linear regression demonstrated excellent agreement between the fluidic-circuit predictions and micro-PIV measurements (*Y* = 1.0607 *X*, R^2^ = 0.9977, and *p* < 0.001). In contrast, the CFD results systematically underestimated the shear rate by approximately 30% relative to micro-PIV measurements, despite maintaining a strong linear correlation (*Y* = 0.7001 *X*, R^2^ = 0.9977, and *p* < 0.001). These results validated the fluidic circuit analysis as a simple and reliable tool for estimating shear rates throughout the microfluidic network and selecting an appropriate *Q_sp_* to establish the shear conditions required for RBC aggregation.

### 3.2. Impact of Supplied Flow Rate on Spatiotemporal AI Map

Because RBC aggregation is strongly affected by shear rate, the effect of flow rate on the RBC aggregation index (AI) was quantitatively evaluated. To promote RBC aggregation, the test blood (Hct = 50%) was prepared by suspending normal RBCs into a dextran solution (30 mg/mL). Based on the shear rate estimates shown in Figure 5, the test blood was infused at flow rates ranging from 0.5 to 5 mL/h.

**Figure 6:**
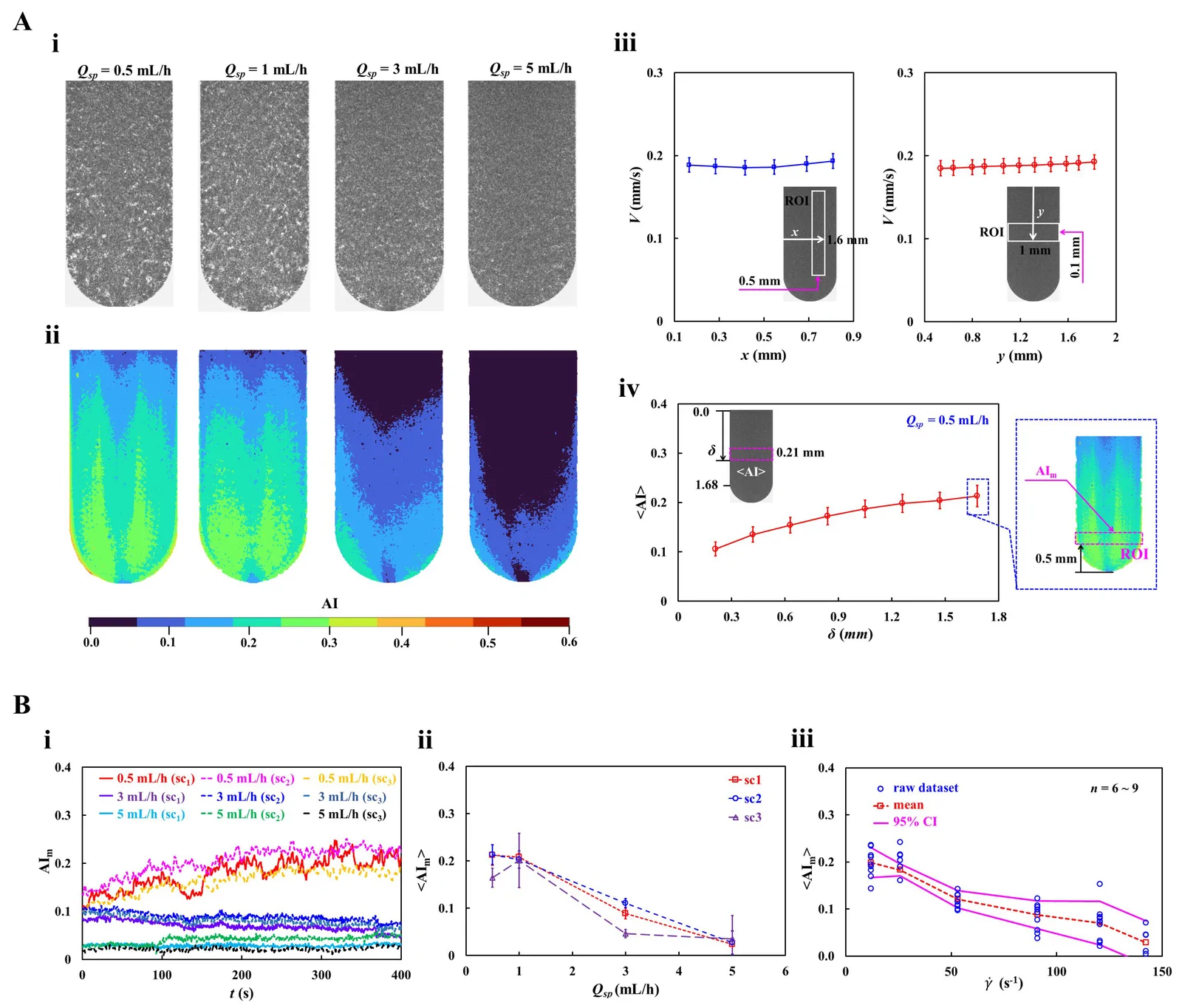
Impact of supplied flow rate on spatiotemporal AI map. Herein, test blood (Hct = 50%) was prepared by mixing normal RBCs into a dextran solution (30 mg/mL). The blood flow rate was set to *Q_sp_* = 0.5~5 mL/h. (**A**) Determination of a proposed index (AI_m_) with respect to downstream distance. (**i**) Microscopic images as a function of *Q_sp_* = 0.5, 1, 3, and 5 mL/h. (**ii**) Spatial AI maps with respect to *Q_sp_*. (**iii**) Velocity distributions in side chambers. The left panel shows variations in *V* along a channel width direction (*x*). The right panel presents the variations in *V* along a flow movement direction (*y*). (**iv**) Determination of representative index (AI_m_). The left panel shows variations in <AI_m_> along the flow direction. The right panel indicates the location of AI_m_, where <AI> reached its maximum value. (**B**) Effect of shear rates on AI_m_. (**i**) Time-lapse AI_m_ in each side chamber as a function of *Q_sp_* = 0.5, 3, and 5 mL/h. (**ii**) Variations in AI_m_ in each side chamber (sc_1_, sc_2_, and sc_3_) with respect to *Q_sp_* = 0.5, 1, 3, and 5 mL/h. (**iii**) Variations in AI_m_ as a function of $\dot{\gamma }$. Repeated measurements were performed with *n* = 6~9 replicates.

As shown in Figure 6A(i), microscopic images were captured at *Q_sp_* = 0.5, 1, 3, and 5 mL/h. The image intensity decreased markedly as infused flow rate increased from 0.5 mL/h to 5 mL/h. Using the definition of AI, as shown in Figure 6A(ii), spatial maps of AI were generated at different *Q_sp_* values. At low flow rates of *Q_sp_* = 0.5 and 1 mL/h, the test blood exhibited higher AI values. Moreover, the AI gradually increased along the flow direction up to the entrance of the downstream side-branch channel. Figure 6A(iii) presents the velocity distributions within a side chamber in the transverse (*x*) and streamwise (*y*) directions. The left panel shows the velocity profile across the channel width, measured using an ROI of 0.8 mm^2^. The right panel shows the velocity variation along the flow direction using an ROI of 0.1 mm^2^. The results indicated that the velocity remained nearly uniform throughout the side chamber. As shown in Figure 6A(iv), a representative aggregation index (AI_m_) was determined from the spatial AI distribution along the flow direction. The left panel presents the variation in the spatially averaged aggregation index: ⟨AI⟩ with the downstream position (*δ*) at *Q_sp_* = 0.5 mL/h. The analysis was performed using a fixed ROI of 0.21 mm^2^. RBCs enter each side chamber in a relatively disaggregated state after passing through the upstream narrow channel. As they traveled downstream through the low-shear chamber, RBC aggregation progressively developed with increasing residence time. Consequently, the spatially averaged AI increased along the flow direction and reached its maximum at δ = 1.68 mm. Thus, the observed AI distribution was primarily associated with progressive RBC aggregation under low shear rather than flow recirculation. As shown in the right panel, the <AI> reached its maximum value at approximately 0.5 mm downstream of the bifurcation entrance. The value of <AI> at *δ* = 1.68 mm was then defined as AI_m_. The time-dependent AI_m_ was evaluated at different imposed flow rates (*Q_sp_*). Figure 6B(i) shows the temporal variations in AI_m_ for the three side chambers at *Q_sp_* = 0.5, 3, and 5 mL/h. In each side chamber, AI_m_ remained nearly constant over time and exhibited its highest value at the lowest flow rate. To quantify the effect of *Q_sp_*, AI_m_ was averaged over time and denoted as <AI_m_>. Figure 6B(ii) presents <AI_m_> for each side chamber (sc_1_, sc_2_, and sc_3_) at *Q_sp_* = 0.5, 1, 3, and 5 mL/h. The <AI_m_> values remained nearly unchanged between 0.5 and 1 mL/h but decreased markedly when *Q_sp_* exceeded 1 mL/h. Figure 6B(iii) shows the relationship between <AI_m_> and shear rate, based on 6~9 repeated measurements (*n* = 6~9). The results demonstrated that <AI_m_> decreased substantially with increasing shear rate.

From the experimental investigation, the spatiotemporal AI maps showed high and stable RBC aggregation at low flow rates (*Q_sp_* = 0.5~1 mL/h). Above 1 mL/h, AI decreased markedly because of the increased shear rate. Based on these experiments, for the consistent measurement of spatiotemporal AI maps, an infusion flow rate was set at *Q_sp_* = 1 mL/h for stable, spatially resolved RBC aggregation measurements.

### 3.3. Contribution of Blood Medium to Spatiotemporal AI Map

Dextran is widely used as a model macromolecular suspending medium to induce RBC aggregation in vitro [44,45,46,47,48]. Because RBC aggregation depends on both the concentration and molecular weight of dextran, samples with different aggregation levels can be prepared by varying the dextran concentration [49]. Based on previous microfluidic studies [24,33,50], test blood (Hct = 50%) was prepared by suspending normal RBCs in dextran solutions (*C_dex_* = 5~40 mg/mL). The test blood was infused at *Q_sp_* = 1 mL/h, which provided sufficiently low shear rates to induce RBC aggregation in the side chambers.

**Figure 7:**
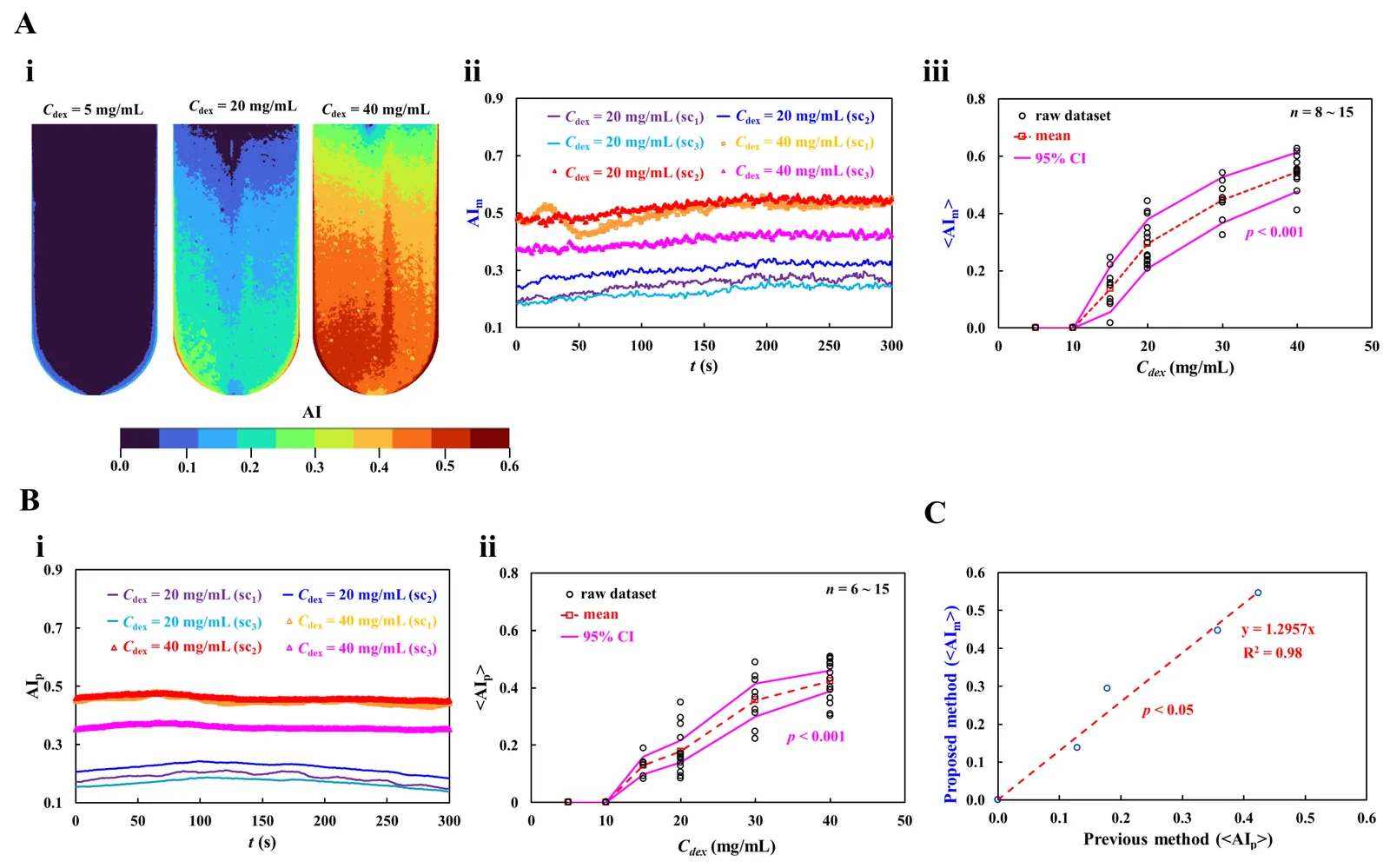
Contribution of dextran solution to spatiotemporal AI maps. Herein, test blood (Hct = 50%) was prepared by mixing normal RBCs into varying dextran solution (*C_dex_* = 5~40 mg/mL). The flow rate of test blood was fixed at *Q_sp_* = 1 mL/h. (**A**) Contribution of dextran solution to AI_m_. (**i**) Spatial AI map with respect to *C_dex_* = 5, 20, and 40 mg/mL. (**ii**) Time-lapse AI_m_ in each side chamber (sc_1_, sc_2_, and sc_3_) with respect to *C_dex_* = 20 and 40 mg/mL. (**iii**) Variations of <AI_m_> with respect to *C_dex_* = 5, 10, 20, 30, and 40 mg/mL. Repeated measurements were performed with *n* = 8~15 replicates. (**B**) Variations in previous AI (AI_p_) as a function of *C_dex_*. (**i**) Time-lapse AI_p_ in each side chamber with respect to *C_dex_* = 20, and 40 mg/mL. (**ii**) Variations of <AI_p_> with respect to *C_dex_*. Repeated measurements were performed with *n* = 6~15 replicates. (**C**) Quantitative comparison between proposed method (<AI_m_>) and previous method (<AI_P_>).

The effect of dextran concentration (*C_dex_*) on AI_m_ was quantitatively evaluated. Figure 7A(i) showed the spatial AI distributions in the side chambers at *C_dex_* = 5, 20, and 40 mg/mL. At *C_dex_* = 5 mg/mL, AI remained close to zero, indicating negligible RBC aggregation. At *C_dex_* = 20 and 40 mg/mL, AI increased markedly and gradually rose along the flow direction. The AI_m_ value was extracted from each spatial AI map at every measurement time. Figure 7A(ii) presents the temporal variations in AI_m_ for the three side chambers (sc_1_, sc_2_, and sc_3_) at *C_dex_* = 20 and 40 mg/mL. Higher concentrations of dextran solution produced substantially greater AI_m_ values, which remained stable over time. The time-averaged value, denoted as <AI_m_>, was then calculated for each concentration of dextran solution. Figure 7A(iii) shows <AI_m_> at *C_dex_* = 5, 10, 20, 30, and 40 mg/mL. Herein, 8~15 repetitive measurements for each concentration were carried out (*n* = 8~15). The <AI_m_> values remained close to zero at 5~10 mg/mL. However, they increased significantly at *C_dex_* ≥ 15 mg/mL (*p* < 0.001). The increase in <AI_m_> above *C_dex_* = 15 mg/mL agreed with previous findings showing that dextran-induced RBC aggregation or sedimentation becomes pronounced at intermediate dextran concentrations [44,49,51,52].

The previously reported method [31] was used as a reference for comparison with the spatiotemporal AI method proposed in this study. Although the previous method enabled RBC aggregation to be measured under continuous blood flow, it quantified aggregation using the mean image intensity over the entire side chamber rather than its spatial distribution. The mean intensity in the main channel was used as the reference value. Accordingly, the conventional aggregation index (AI_p_) was calculated as AI_p_ = (*I_mc_* − *I_sc_*)/*I_mc_*, where *I_mc_* and *I_sc_* represent the mean image intensities of the main channel and side chamber, respectively.

As shown in Figure 7B(i), the temporal variations in AI_p_ were measured in each side chamber at *C_dex_* = 20 and 40 mg/mL. The AI_p_ values showed trends similar to those of AIm. The time-averaged AI_p_, denoted as <AI_p_>, was then calculated for each dextran concentration. Figure 7B(ii) shows that <AI_p_> increased markedly at *C_dex_* ≥ 15 mg/mL. To compare the two indices, <AI_m_> was plotted against <AI_p_> at the corresponding dextran concentrations. Linear regression yielded <AI_m_> = 1.2957<AI_p_> with R^2^ = 0.98 and *p* < 0.05. Thus, the two indices were strongly correlated, while <AI_m_> was approximately 30% higher than <AI_p_>, indicating a stronger response to dextran-induced RBC aggregation. The strong linear correlation between **<**AI_m_> and **<**AI_p_> confirmed that both indices reliably represented dextran-induced RBC aggregation. This finding was consistent with previous studies using microscopic image intensity to quantify RBC aggregation in microfluidic channels [22,53]. However, the <AI_m_> was approximately 30% higher than <AI_p_> because it was measured in a specific region where aggregation was most pronounced, whereas <AI_p_> was calculated by averaging the intensity over the entire side chamber. Whole-chamber averaging may weaken localized intensity changes caused by RBC aggregation. Therefore, the proposed spatiotemporal method provides improved AI measurement values while revealing spatial variations in RBC aggregation that cannot be identified using a single chamber-averaged index.

From the experimental results, RBC aggregation was negligible at *C_dex_* = 5~10 mg/mL but increased markedly above 15 mg/mL. The measured AI_m_ was approximately 30% higher than the conventional AI_p_ under strongly aggregating conditions.

### 3.4. Impact of Storage Duration on Spatiotemporal AI Map

As a final demonstration, the proposed method was used to evaluate the effect of RBC storage duration on spatiotemporal AI maps. Previous studies have reported storage-dependent changes in RBC aggregability, including reduced aggregation under certain storage and measurement conditions [54,55,56,57,58,59]. To examine whether the proposed method could detect these changes, test blood (Hct = 50%) was prepared by suspending stored RBCs in dextran solutions with *C_dex_* = 15~40 mg/mL.

**Figure 8:**
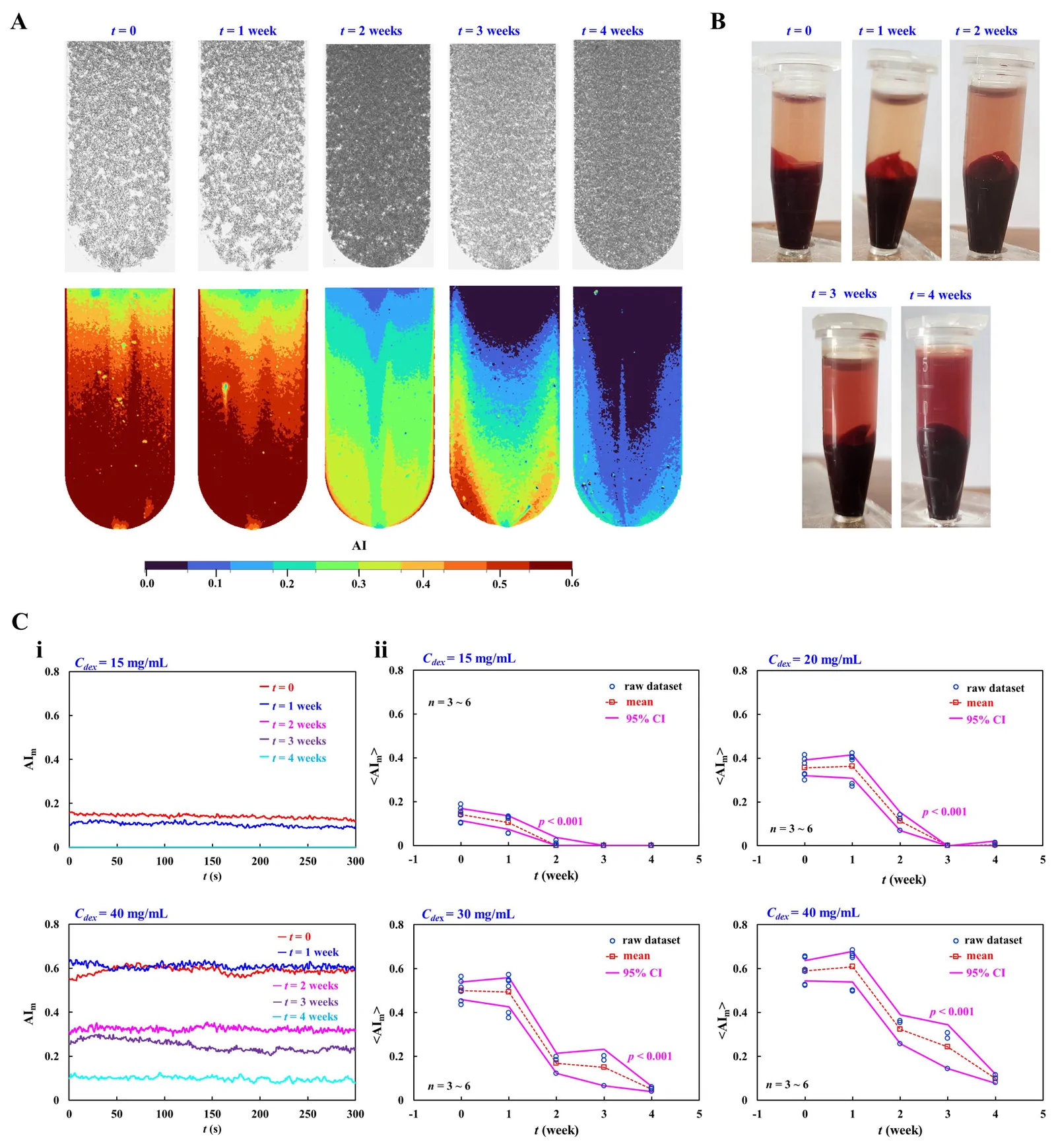
Impact of storage duration on spatiotemporal AI maps. (**A**) Spatial heterogeneity of AI over storage duration (*t* = 0, 1, 2, 3, and 4 weeks). The upper panel shows microscopic images for test blood after 0, 1, 2, 3, and 4 weeks of storage. The lower panel exhibits a spatial mapping of AI with respect to storage duration. (**B**) Snapshots of tubes filled with test blood after centrifugation as a function of storage duration. (**C**) Contribution of storage time and dextran solution to <AI_m_>. Repeated measurements were performed with *n* = 3~6 replicates. (**i**) Time-lapse AI_m_ of test blood with respect to storage time and dextran solution (*C_dex_* = 15 and 40 mg/mL). (**ii**) Variations in <AI_m_> with respect to storage time and dextran concentration (*C_dex_* = 15, 20, 30, and 40 mg/mL).

As shown in Figure 8A, microscopic images and spatial AI distributions were obtained at different storage times. Test blood (Hct = 50%) was prepared by suspending normal RBCs in a dextran solution (40 mg/mL). The upper panel presents representative microscopic images acquired after 0, 1, 2, 3, and 4 weeks of storage, while the lower panel shows the corresponding spatial AI distributions. The AI decreased markedly with increasing storage time, indicating a progressive reduction in RBC aggregation. To assess potential RBC damage, the color of the suspending medium was examined after centrifugation. As shown in Figure 8B, no noticeable color change was observed during the first week. However, after two weeks of storage, the suspending medium became progressively redder, consistent with storage-induced hemolysis and the accumulation of extracellular hemoglobin released from damaged RBCs [60,61,62].

As shown in Figure 8C(i), the temporal variations in AI_m_ were measured over 0~4 weeks of storage at *C_dex_* = 15 and 40 mg/mL. At *C_dex_* = 15 mg/mL, the AI_m_ showed little change during the first week. However, at *C_dex_* = 40 mg/mL, the effect of storage time was more clearly observed. The time-averaged value, denoted as <AI_m_>, was then calculated for each storage time. Figure 8C(ii) presents the variation in <AI_m_> over 0~4 weeks at dextran concentrations ranging from 15 to 40 mg/mL. Repeated measurements were performed at each storage time (*n* = 3~6). The <AI_m_> values were substantially higher at higher dextran concentrations but decreased markedly with increasing storage time (*p* < 0.001). The marked decrease in <AI_m_> with storage time indicated a progressive loss of RBC aggregability, consistent with previous studies using conventional and microfluidic aggregation measurements [54,55,56,58]. Because the dextran concentration was fixed within each experimental series, this decrease was mainly associated with storage-induced cellular changes rather than changes in the suspending medium. Prolonged storage caused ATP depletion, membrane damage, hemolysis, and morphological transformation of RBCs from discocytes into echinocytes or spherocytes, which could reduce their ability to form stable rouleaux [63]. The higher <AI_m_> observed at *C_dex_* = 40 mg/mL provided a wider measurement range, allowing storage-dependent changes to be detected more clearly than at *C_dex_* = 15 mg/mL.

From the demonstration results, RBC aggregation decreased markedly with increasing storage duration, indicating a progressive loss of RBC aggregability. The proposed method successfully detected these storage-induced changes, particularly at the higher dextran concentration. As a limitation of the present study, first, the experiments used dextran-based RBC suspensions rather than whole blood. The reported AI values were obtained at Hct = 50% and *C_dex_* = 5~40 mg/mL. Therefore, absolute AI values may differ in whole blood due to plasma proteins and other cellular components. Second, only one microfluidic geometry was tested. The bifurcation-channel resistance (12.61~13.87 TPa·s/m^3^) was about 20 times higher than that of the main-channel segments (0.15~0.54 TPa·s/m^3^), producing the required low-shear condition in the side chambers. Future studies should validate the method with whole blood and different channel geometries. Third, the present top-view imaging method did not resolve RBC aggregation along the channel depth. The measured AI represented a depth-integrated and ROI-averaged aggregation response rather than a local three-dimensional value. Future studies using confocal or other depth-resolved imaging techniques will be required for a three-dimensional characterization of RBC aggregation.

## 4. Conclusions

This study proposed a microfluidic method for the spatiotemporal measurement of RBC aggregation under continuous blood flow. Multiple high-resistance side chambers produced low shear-rate conditions for RBC aggregation, while the main channel maintained a sufficiently high shear-rate to disperse RBCs. Analytical calculations, numerical simulations, and micro-PIV measurements confirmed the flow rates and shear rates in the microfluidic channels. The spatiotemporal AI maps showed that RBC aggregation remained high and stable at *Q_sp_* = 0.5~1 mL/h. However, it decreased markedly at higher flow rates. The proposed method was further demonstrated using different dextran concentrations and RBC storage durations. RBC aggregation increased markedly at *C_dex_* ≥ 15 mg/mL, and the proposed AI_m_ showed an approximately 30% greater response than the conventional AI_p_. In addition, AI_m_ decreased progressively over four weeks of storage, indicating a storage-dependent loss of RBC aggregability. These findings demonstrated that the proposed method could sensitively visualize and quantify the spatial and temporal variations in RBC aggregation without interrupting blood flow. Therefore, this method could provide a useful platform for continuous monitoring of RBC aggregation and storage-related changes in blood quality.

## Data Availability

The original contributions presented in this study are included in the article. Further inquiries can be directed to the corresponding author.
